# Dietary Phenolic Compounds—Wellbeing and Perspective Applications

**DOI:** 10.3390/ijms25094769

**Published:** 2024-04-27

**Authors:** Dasha Mihaylova, Maria Dimitrova-Dimova, Aneta Popova

**Affiliations:** 1 Department of Biotechnology, Technological Faculty, University of Food Technologies, 4002 Plovdiv, Bulgaria; 2 Department of Catering and Nutrition, Economics Faculty, University of Food Technologies, 4002 Plovdiv, Bulgaria; mcollege_plovdiv01@abv.bg

**Keywords:** biologically active compounds, health benefits, prevention through nutrition, plant diet, environmental challenges

## Abstract

Contemporary living is continuously leading to poor everyday choices resulting in the manifestation of various diseases. The benefits of plant-based nutrition are undeniable and research on the topic is rising. Modern man is now aware of the possibilities that plant nutrition can provide and is seeking ways to benefit from it. Dietary phenolic compounds are among the easily accessible beneficial substances that can exhibit antioxidant, anti-inflammatory, antitumor, antibacterial, antiviral, antifungal, antiparasitic, analgesic, anti-diabetic, anti-atherogenic, antiproliferative, as well as cardio-and neuroprotective activities. Several industries are exploring ways to incorporate biologically active substances in their produce. This review is concentrated on presenting current information about the dietary phenolic compounds and their contribution to maintaining good health. Additionally, this content will demonstrate the importance and prosperity of natural compounds for various fields, i.e., food industry, cosmetology, and biotechnology, among others.

## 1. Introduction

The health consequences of diseases are amplified by poor nutrition. Poor nutrition can be defined as a major societal challenge. On the one hand, various populations do not have access to nutritious food and clean water, while on the other, many are exposed to excessive amounts of refined foods and sugar-added drinks that negatively influence their overall health. The dynamics of global nutrition are marked by foods high in salt, sugars, and fats, which result in the manifestation of overweight and obesity, as well as type II diabetes and cardiovascular abnormalities [1]. Oxidative stress, or simply the imbalance between oxidants and antioxidants, with oxidants prevailing, leads to a cascade of reactions in the organism in favor of poor health outcomes [2]. Additionally, several environmental factors contribute to the worsening of overall health. Pollution of various kinds exposes human beings to harmful environments on a daily basis. Microplastic pollution is seen as one of the major threats to ecosystems and humans [3], which has a proven negative effect on living organisms [4]. Environmental contaminants substantially contribute to human disease, disturbing public and individual health and resulting in rising mortality and morbidity [5]. Countless developed countries are on top of the list when it comes to first harming and consequently introducing preventive strategies [5].

Many studies present the connection between food consumption and the content of bioactive compounds during the past years. Phenolic compounds are among the most studied in terms of not only the color and taste of food components but also providing complimentary health benefits to their consumers [6]. Wellbeing associated with disease-preventive nutrition has been a trending topic for more than 10 years [7]. Fruits, vegetables, and plants, in general, are known sources of biologically active compounds that supplement the daily human diet [8,9]. Plant nutrition is notoriously recognized for its preventive role in the occurrence of common chronic diseases like diabetes, obesity, and hypertension. In this perspective, microgreens are innovative sources of physiologically active compounds with highly valuable effects [10]. Microgreens consist of vegetables, grains, and herbs with appealing sensory (miniature size and tender structure) and favorable nutritional qualities [7]. Microgreens are considered the next generation of superfoods with their abundant level of various phytonutrients, i.e., polyphenolic compounds [11]. Other polyphenol-rich dietary products include tea, coffee, cocoa-based products, wine, cereals, herbs and spices, nuts, and seeds [12,13,14]. Polyphenolic compounds are naturally synthesized by secondary metabolic systems in plants and primarily gained researchers’ interest because of their antioxidant effect. The antioxidant, antiatherogenic, anti-inflammatory, antimicrobial, antithrombotic, cardioprotective, and vasodilator properties [15,16] are only a few of the biological effects of phenolic compounds. Currently, it was found that dietary polyphenols can modulate the composition of intestinal microbes [17]. Polyphenols can also adjust some gut microbiota metabolites (short-chain fatty acids, dopamine, bile acids, and lipopolysaccharides) [18]. In this view, the association between diet, health, and the presence of bioactive compounds in food has received great attention in recent years [8,19]. However, it has to be noted that along with all positive effects, plant-based nutrition can exert a list of antinutritional effects due to the presence of phytates, oxalates, goitrogens, saponins, oxalates, and lectins [20]. Many papers suggest that the phytochemical content of plants depends on the origin, cultivation, environmental conditions, ripeness, pre- and post-harvest storage, along with transportation and plant part being used (seeds, fruits, leaves, and stem) [21,22].

Different approaches are pursued in the extraction of phenolic compounds. Depending on their solubility and attachment to other biological molecules, phenolic compounds can be seen as free and bonded [23]. The solvent choice is an important step in polyphenol extraction. The commonly applied methods are alkali and acidic extractions [24]. Other methods include heat application, microwave or ultrasound extractions, along with enzymatic treatment procedures [25]. Currently, a unified most suitable extraction technique has not been found [26].

This review is focused on providing current information about the dietary phenolic compounds and their contribution to maintaining good health. Additionally, the presented information will demonstrate the importance and prosperity of natural compounds for various fields, i.e., the food industry, cosmetology, and biotechnology, among others.

## 2. Dietary Phenolic Compounds

**Figure 1:**
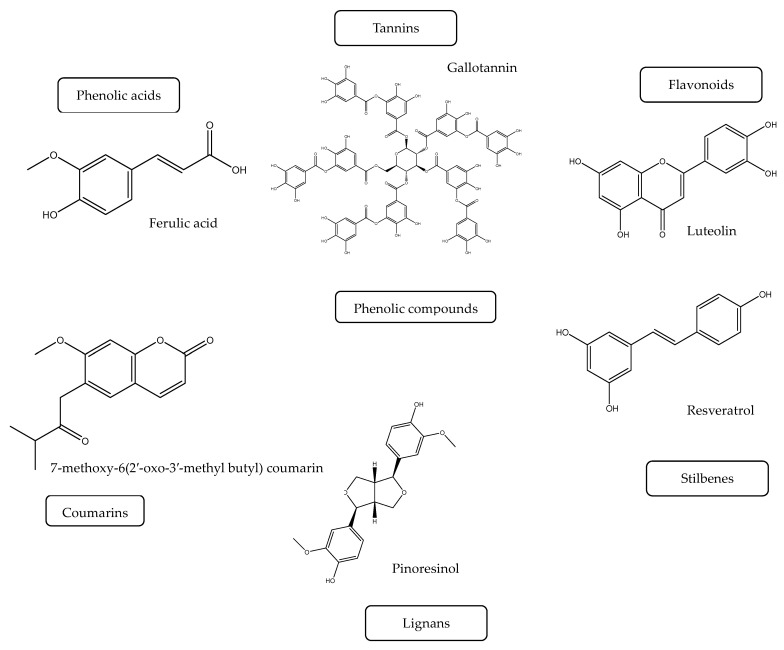
Some phenolic compound representatives.

Dietary phenolic compounds have large chemical variability, more than 8000 phenolic structures have been described and categorized into several classes [12]. Phenolic compounds (Figure 1) are dependent on their structure and have a variety of structures, including phenolic acids, flavonoids, stilbenes, tannins, coumarins, and lignans [6,27]. Some researchers determine food bioactive compounds as all compounds found naturally in food that can provide a certain bioactive effect on the human body but are mostly without any nutritional value [15]. The classification of these compounds is performed based on their chemical structure, composition, and their synthetic pathways. Dietary polyphenols represent a wide range of secondary metabolites, primally derived from phenolic acid, catechins, flavones, and isoflavones [6,28].

Araújo et al. divide phenolic compounds into the following groups—flavonoids, phenolic acids, anthocyanins, and tannins [8]. Camara et al. propose a major food bioactive compounds classification based on the food source where dietary phenolic compounds can be: phenols (chlorogenic acid in blueberry and raspberry fruits), phytosterols (stigmasterol in soybean), terpenoids (limonene in citrus fruits), polysaccharides (cellulose in flax seeds), carotenoids and tocopherols (β-carotene/vitamin A), glucosinolates (sulforaphane in broccoli), triterpenes (squalene from olive oil), alkaloids (caffeine in coffee beans), capsaicinoids (capsaicin in peppers), bioactive peptides (carnosine in red meat), and PUFAs (polyunsaturated fatty acids, docosahexaenoic acid—DHA). Polyphenols can also be divided into two main groups: flavonoids and non-flavonoids [15,29].

### 2.1. Phenolic Acids

Phenolic acids are fundamental dietary components. They are natural compounds that possess one carbohylic acid group [30]. There are two sub-groups of phenolic acids, representing hydroxylated derivatives, respectively, of benzene (hydroxybenzoic) and cinnamic (hydroxycinnamic) acids. Chlorogenic acid is a common hydroxycinnamic acid, along with ferulic, caffeic, *p*-coumaric, and sinapic acids. Vanillic, *p*-hydroxybenzoic, protocatechuic, and syringic acids have recently been identified as hydroxybenzoic acids [28]. Phenolic acids are synthesized through the phenylpropanoid pathway and can be found in free, soluble, conjugated, and insoluble forms [31]. Caffeic and ferulic acids are widely metabolized in the human body after their absorption in the gastrointestinal tract [32]. Caffeic acid has been reported to positively influence various types of cancer, as well as diabetes, obesity, and metabolic syndrome [33]. A recent review of the literature highlights that mixtures of phenolic acids are efficient bioactive dietary ingredients [34]. Plants usually contain several phenolic acids in them [35]. Research suggests that people can benefit from plant phenolics due to their various activities (skin anti-aging, disease management, and tissue damage control, among others) [26].

### 2.2. Flavonoids

Flavanones, flavones, isoflavones, flavonols, flavanols, and anthocyanins form the seven classes of flavonoids based on their structure [36]. Flavonoids represent one of the most important and numerous subgroups of natural phenols. They significantly contribute to the color and aroma of many fruits (berries, grapes, apples, and others) and vegetables (onions, cabbage, and others). The phenylpropanoid pathway is mainly responsible for the biosynthesis of flavonoids [37]. Most flavonoids are found in leaves, flowers, fruits, and seedlings [38]. They are known for their cell-signaling, anti-thrombogenic, and neuroprotective properties [28,29]. The established properties (anti-inflammatory, antiviral, antiallergic, antihypertensive, anticarcinogenic, and hepatoprotective) of flavonoids set a path for further plant studies that can possibly identify new structures and pathways various industries could benefit from [39]. Due to flavonoids having the same skeleton, it is the replacement groups that are mainly responsible for their functional differences [40]. A broad range of health-revitalizing effects is attributed to anthocyanins [41].

### 2.3. Lignans

Lignans in plants are a group of natural compounds formed from two units of phenylpropane. They are important for plants’ defense strategies [42]. Some authors suggest that lignans are widespread in the plant kingdom, mostly in their free state rather than in the form of glycosides [43]. Lignans are described in eight subtypes and are also diversified based on the presence of oxygen [44]. Lignans accumulate in all plant organs, but most are contained in the seeds, fruits, roots, and barks [45]. Flaxseed is the richest lignan source in the human diet [46]. Other sources include wheat, lentils, pears, prunes, garlic, asparagus, carrots, sesame, etc. [15]. Lignans have gained sizable researchers’ interest due to their pharmacological activities [47].

### 2.4. Stilbenes

Stilbenes can be observed in their free, glycosylated, prenylated, and methoxylated forms and are characterized by a variety of chemical compounds [48]. Stilbenes are present in small amounts in plant organs and tissues. Their synthetic pathway can differ, with stilbene synthase playing a key enzyme role [49,50]. A wide variety of representatives with various biological activities exist. Resveratrol in its isomeric, trans, and glycosylated forms is probably the most spread and well-known [51]. In plants, its major form is trans-resveratrol-3-*O*-β-d glycoside. Piceatannol, found in almonds, peanuts, teas, grapes, berries, and passion fruit, is a structural analog associated with resveratrol that exhibits a variety of health-promoting biological properties (antioxidant, anti-inflammatory, and anticancer) [52]. In recent years, researchers’ attention has been focused on the structural classifications and pharmacological activities of stilbenes [53].

### 2.5. Tannins

Plant tannins possess a diverse structure, but they are generally divided into hydrolyzed and condensed tannins [54]. Tannins are seen as promising antibacterial and antivirulence agents for the avoidance of bacterial infections [55]. Tannin-rich plant extracts are reported to have anti-hypercholesterolemia, anti-diabetic, antioxidant, anticancer, and antimicrobial activities [56].

### 2.6. Coumarins

Coumarins can be found in various plant sources like roots, leaves, flowers, and fruits [57]. Chemically, they belong to the lactones family [57]. Coumarins can be classified as simple, furano-, pyrano-, dihydrofyrano-, phenyl-, and bi-coumarins [57]. They can be used as an inflammation treatment, anticoagulants, antioxidants, and enzyme inhibitors [58]. There is evidence of the potential of coumarins in the management of degenerative diseases like Alzheimer’s and Parkinson’s [59]. However, their presence in various food sources is carefully studied, and even some restrictions on a maximum daily intake exist [60]. Additionally, there are some reported cases of hepatic and pulmonary toxicity [61].

Table 1 gives a summary of some of the widespread dietary phenolic compounds, highlighting their ability to manage various diseases and pointing to the appropriate food sources.

## 3. Current Applications

Dietary phenolic compounds’ bioavailability is influenced by different factors and processes such as interaction with other compounds, concentrations in the food, molecular size, release in the food matrix, chemical structure, degree of polymerization and solubility, digestion, absorption, and metabolism [15]. The efficient extraction of polyphenols from plant matrices makes them a basis for further study and practical applications [73].

### 3.1. Bioactive Packaging, Coatings, and Preservatives

Food packaging is an effective way of protection and shelf life extension [74]. However, the use of chemicals in packaging can be harmful to both the environment and living beings. In this view, it became necessary to provide innovative packages with bioactive components in them [14]. Polyphenolic extracts can successfully be used for the preparation of bioactive packaging and coatings [75]. One of the most important advantages of bioactive packaging is that it is perceived as eco-friendly and economically valuable for the food sector [76]. Some researchers report that food packaging from gelatin-based films with extracts containing dietary phenolic compounds could protect food production for up to 30 days [22]. Edible coating and film formulations are used to increase biodegradability and to reduce pollution caused by environmental waste. Edible coatings can help improve food quality and prevent oxidation and color change under different environmental effects. The presence of coating/film or active packaging is proven to prevent volume reduction and the associated increase in apparent density, improving the physical properties of food during storage [77]. Successful research designs showed that coating can enhance the surface color of dried fruits [78]. Edible coatings are effective for preventing the oxidation of bioactive compounds. Researchers found that the total phenol content of coated samples was higher than that of uncoated ones [14,79].

Active food packaging has focused its attention on bio-based functional packaging materials containing natural active compounds and ingredients. Different incorporation mechanisms are currently being used: the addition of emitting sachets, absorbent pads, dispersion of phenolic compounds in the packaging polymer, coating, or dipping [80]. A recent innovation involves a color-changing wrap that indicates when food has gone bad [81]. The use of natural additives in the food industry has increased in the last decades due to their beneficial effects on food preservation (antioxidant/antimicrobial properties), as well as due to the consumers’ demand for natural food ingredients [82]. The food industry is constantly seeking to discover antimicrobial agents that can prevent food spoilage and, as a result, increase the safety and shelf life of the final food product. This is the reason to include polyphenol agents in packaging. Food packaging based on natural materials with proven antimicrobial effects could reduce pathogen microorganisms and extend food shelf life [19,83].

Dietary phenolic compounds have potential use as biopreservatives in the food industry. In fact, phenolic compounds have been extensively studied for their application in the food industry for improving the shelf life of perishable products and allowing the production of food without synthetic additives for consumers because the current concern about the impact of food on health has been influencing the consumer choice of food based on its formulation [80].

### 3.2. Natural Colorants

The re-introduction of natural dying sources derives from the various harmful effects (allergic, toxic, and carcinogenic responses) synthetic (mostly based on petroleum) dyes exert on living beings [84]. Color additives are fundamental for the food industry, as well as for the production of textiles and cosmetics, among others [85]. During food storage, a significant amount of its original color can be lost. This “defect” can be eliminated by using natural or synthetic food dyes. Natural dyes have antimicrobial, antioxidant, and therapeutic properties against diseases and health disorders [86]. Different countries have different laws and restrictions that apply to dying agents. The Food and Agriculture Organization (FAO)/World Health Organization (WHO) Expert Committee on Food Additives is the international body responsible for evaluating the safety of food additives; the Food and Drug Administration (FDA) is operating in the United States of America (USA), while in the European Union (EU), it is the European Food Safety Authority (EFSA) that is locally responsible for controlling safety.

Anthocyanins, carotenoids, phenolic compounds, red beet derivatives, chlorophylls, and some curcuminoids are some of the most widely used natural dyes [87]. Natural dyes, along with their positive features (bioactive molecules and antioxidant effects), can exhibit some negativities as well, i.e., hyperactivity in children, contaminants, or pesticide residues [88]. Some of the popular and common natural polyphenolic colorants include anthocyanins and tannins. Tannins are studied as a renewable natural pigment in liquid, paste, and dry forms [89]. Ellagitannins are reported as important for the color of wine during aging [90]. Flavonoids appear in nature from the orange-red to purple-blue spectra [91]. Additionally, natural sources like turmeric (orange), beetroot (red), and spirulina (green) manifest not only their health-promoting properties but also their distinguishable vivid colors [92].

### 3.3. Antioxidant, Antimicrobial and Antiviral Activities

The antioxidant activity of polyphenols, along with various extraction techniques, has been studied for years [93]. The antioxidant capacity of polyphenols differs by their chemical structure, stability, and bioavailability, among others. Polyphenols are associated with reactive oxygen species removal and metal ion chelation [94]. However, it has to be noted that polyphenols exhibit their antioxidant effect at low concentrations, while at higher concentrations, they can have a pro-oxidant effect [94]. The abovementioned suggests that the positive or negative influence of polyphenols is highly dose-dependent. Polyphenols are described as active substances against various types of viral infections like influenza, hepatitis, herpes, rotavirus, and even coronavirus [95]. The mechanism of action of each polyphenolic compound is not the same; thus, the mechanism behind any virus inhibition depends on the specificity of the virus and the given polyphenolic compound [96]. Some of the most documented polyphenol compounds with antiviral activity include catechin, tannic acid, gallic acid, resveratrol, kaempferol, and quercetin [97,98]. Hesperidin was reported as a possible strategy against SARS-CoV-2 [99]. The growth cycle of some viruses can be inhibited by quercetin, gallic acid, and epigallocatechin [100].

The lack of new antibiotics and antibiotic resistance makes it mandatory to find new strategies against both Gram-positive and Gram-negative pathogenic microorganisms [100]. Plants have long been used in the treatment of infections caused by bacteria due to the presence of biologically active compounds in them [101]. Polyphenols have noteworthy antimicrobial activity, but their properties are affected by the bacterial cell structure difference, extraction variations of polyphenolic compounds, and the exposure duration of microorganisms to polyphenols [102]. Recent research suggests that natural phenolic compounds are promising candidates for microbial therapy, but several aspects should still be clarified regarding the structure-function relationships [103]. Epicatechin gallate and (-)-epigallocatechin gallate are reported to enhance the effect of antibiotics [104]. Future findings on this topic may help with the rising list of multi-drug-resistant organisms.

### 3.4. Developing New Food Products with Enhanced Polyphenol Content

Many consumers are searching for food products with enhanced content. This applies to not only polyphenols but also other compounds with beneficial properties.

For example, the practice of transforming plants and fruits in other derived foodstuffs following fermentation has been used far away in history. Nowadays, fermentation is looked at as a way to provide health benefits due to the functional activity of the fermented produce [105]. Additionally, spontaneous or probiotic fermentation shows a higher bioavailability of phenolic compounds [106].

Due to the existing antimicrobial activity of polyphenols (see Section 3.3), extracts or freeze-dried plant powders become parts of recipes in order to provide better health and nutritional outcomes for the consumer [107,108]. Furthermore, several by-products like peels and kernels can be utilized as value-added ingredients to different baked goods [109]. The existing demand for polyphenol-rich oils is also a topic of continuous research [110].

## 4. Wellbeing and Prevention through Nutrition

Nutrition is related to many aspects of human wellbeing, i.e., health status and mood. Its ultimate goal is to preserve health and wellbeing [111]. There is evidence that certain dietary patterns can affect mental health [112]. Lifestyle changes emphasize many “diseases of civilization” [113]. Healthy eating patterns where fruit and vegetables are included in the diet are associated with better mental health [114]. Additionally, the consumption of anthocyanin-rich fruits has been associated with beneficial effects on the brain through neuroinflammation, neurogenesis, and neuronal signaling modulation [115]. Furthermore, polyphenol supplementation is able to effectively modulate anxiety and depression [116]. Polyphenols are currently stated as potential anti-aging agents because they slow down the shortening of telomeres [117]. Remigante et al. [118] have also suggested that polyphenol-rich extracts prevent the d-galactose-induced radical oxygen species production and thus present a preventive model of aging by human red blood cells. Plant-based diets and those rich in polyphenolic foods or extracts are shown as highly beneficial for health maintenance [119]. The Blue zones confirm these findings by studying the relationship between longevity and the consumption of polyphenol-rich foods, i.e., garden fruits and vegetables, whole-grain breads, and beans [120].

Evolutionary the eating patterns of humans have changed. Researchers suggest that polyphenol intake might have had an important selective role in the development of cognitive abilities [121]. The Mediterranean diet is still stated as the most beneficial in terms of health management and wellbeing [122]. It is associated with the consumption of marine ώ-3 fatty acids, polyphenols from fruit, vegetables, legumes, grains, and nuts, along with olive oil, red wine, and other beneficial substances like minerals, vitamins, and fibers [123]. The consumption of fruit and vegetables mostly contributes to the availability of flavonoids and flavones [124]. Olive oil represents lignans intake, while legumes, gains, and nuts—flavonoids and isoflavonoids [125].

Polyphenols can act synergically in the gut and bloodstream against various biotic and abiotic stressors [126]. Studies support the preventive role of polyphenols in the fight against diseases [127], free radicals, gut health [128], and cholesterol oxidation products [129], among others. Additionally, Xie et al. state that the polyphenols-gut-brain axis may be a key way to regulate glycolipid metabolism [130]. Other authors also report that gut microbiota-polyphenols positively affect human physiological processes [130,131]. Polyphenols not only stimulate the growth of amicable probiotic colonies but also lower the number of pathogenic bacteria [132]. A diet rich in phenolic compounds can lead to qualitative and quantitative diversification of gut microbiota [133]. Polyphenol ingestion has a prebiotic effect on the gut’s host [134]. Dietary phenolic acids have been reported to improve gut function by reducing intestinal inflammation [135]. The consumption of polyphenol-rich food and drink sources may induce positive shifts in gut microbiota composition, as shown by the consumption of polyphenol-rich beers [136]. Polyphenols also have promoting effects on *Akkermansia muciniphila*, an anaerobic and mucosa-associated colonic bacterium with mucin-degrading capabilities [137]. Recent findings conclude that microbial taxa but not diversity are promoted by habitual polyphenol consumption [138]. This implies that future research should focus on specific microbial biomarkers and their changes due to polyphenol exposure.

However, some papers accentuate the negative effects that polyphenols may exhibit on human health. For example, there is evidence of the polyphenols’ ability to block iron uptake, inhibit some digestive enzymes, interact with drugs, and influence hormonal balance [139]. Shaito et al. conclude that although the positive effects of polyphenols are undeniable, their possible harmful nature is still not supported by enough clinical studies [140]. This reminds the need for continuous research on every topic and aspect of human health management.

Microplastic waste in food and water presents a reasonable health concern for living organisms [141]. Microplastics are addressed as a macro issue, and their elimination is an object of current research [142]. Polyphenols can be successfully used for polyethylene terephthalate (PET) elimination due to their ability to interact as hydrogen donors with the carbonyl groups of PET [143]. Creating effective technologies for microplastic elimination is an open topic for further research [144].

## 5. Conclusions and Future Perspectives

People are gradually turning back to natural ingredients, herbal remedies, and traditional medicine that have been used for centuries. Nowadays, there is a trending lifestyle change in terms of the natural. Many drugs contain plant extracts with proven antiviral and antimicrobial activities [145,146]. The existing resistance to antibiotics has led to the seek for old herbal remedies that can manage various diseases [147]. Polyphenols have successfully had their role in maintaing human health and promoting a more conscious lifestyle. Not only food but also everyday cosmetics are returning to all-natural ingredients. Researchers are providing on a daily basis a way to extract and preserve all the beneficial compounds plants can provide. The “green” approach presents ways to turn to a zero waste cycle by using polyphenol-rich by-products like peels, pomace, and kernels, among others [148,149]. A successful microencapsulation of antioxidant compounds was reported by several researchers [150].

In the future, polyphenols should be better described as part of food nutrient information regardless of their zero energy value. They ought to be placed in a separate category as are now vitamins and minerals. Their supplementation should also be well regulated due to their possible harmful effects if taken without consideration.

The understanding of polyphenols’ biological effects, especially when it comes to food, is still not very clear due to the availability of several polyphenol sources in one meal. In order to extend the beneficial effects of polyphenols, their enzymatic interactions and possible synergism or antagonism should be extensively studied. The bioaccessibility of polyphenols after digestion should also be studied and possibly transferred in vivo and to clinical trials.

The sustainability of the plant system should be taken into consideration. The massive demand for plant production can lead to a harmful environmental impact. Plant diversity should be carefully monitored as invasive varieties may overtake the land. This will lead to the loss of important plants rich in biologically active compounds.

## Figures and Tables

**Table 1:** Some dietary phenolic compounds with their respective sources and biological activities.

Class	Dietary Phenolic Compounds	Sources	Biological Activities and Disease Management	Reference
Phenolic acids	Ferulic acid Caffeic acid Gallic acid *p*-Coumaric acid Vanillic acid	oilseeds, cereals, coffee, cowpea, black currant, raspberry, cherries, peaches, blackberry, plums, citrus juices and fruits, squash shells and seeds, spinach, tomatoes, potatoes, and almonds	diabetes (by enzyme inhibition); cancer; neuroprotection; antimicrobial and antiviral properties	[26,62]
Flavonoids	Curcumin Quercetin Rutin Kaempferol Luteolin Cyanidin Catechin Epicatechin	whole grains, coffee, green tea, berries, apple, citrus fruits, tomato, onion, garlic, carrots, cruciferous vegetables (cabbage, broccoli, cauliflower, brussels sprouts)	anti-inflammatory, antiviral, antiallergic, and anticarcinogenic properties; toxin-mediated stress and chronic disease prevention; breast cancer, coronary heart disease, cataracts, diabetes, Alzheimer’s disease	[63,64]
Stilbenes	Resveratrol Pterostilbene ε-Viniferin Raloxifene Tamoxifen	cocoa, grapes, hop, peanut, sugar cane, tomato, bilberry, blueberry, strawberry, mulberry, deerberry	allergies, inflammation of different tissues (cardiac, connective, nervous), intestinal, liver and lung inflammations, enzyme inhibition, obesity	[50,65,66]
Tannins	Gallotannins Ellagotannins	pigeon pea, jack bean, yam bean, babul, black myrobalan, japweed, blackberry, pomegranate, walnut	pathogens control, treatment of diarrhea and skin burn, antioxidant, antimicrobial, anti-inflammatory, and anti-diabetic properties, management of diabetes, obesity, dyslipidemia	[67,68]
Coumarins	Osthole Dicoumarol Thunberginols Psoralen	citrus fruit peels (orange, clementine, lemon), propolis products, oils (olive, soy, peanut, corn), coffee, nuts, wine, green tea, cinnamon	anti-inflammatory, anti-mutagenic, anti-tumorigenic, and antioxidant properties, spasmolysis, inhibition of insulin-induced lipogenesis, antibacterial and anticancer activities	[69,70,71]
Lignans	Sevanol Isoguaiacin Carinol Gomisin	flaxseed, sesame seeds, coffee, tea (black, green), cereals, barley, buckwheat, chickpeas, peas, asparagus, avocado, eggplant, pineapple, oranges, kiwi, lemon, grapes	anti-inflammatory, antioxidant, and antitumor activities, cancer management, cardiovascular disease control, chronic inflammation	[46,72]

## Data Availability

Not applicable.

## References

[1] Muonde M., Olorunsogo T.O., Ogugua J.O., Maduka C.P., Omotayo O., Muonde M., Olorunsogo T.O., Ogugua J.O., Maduka C.P., Omotayo O. (2024). Global Nutrition Challenges: A Public Health Review of Dietary Risks and Interventions. World J. Adv. Res. Rev..

[2] Sies H. (2020). Oxidative Stress: Concept and Some Practical Aspects. Antioxidants.

[3] Anand U., Dey S., Bontempi E., Ducoli S., Vethaak A.D., Dey A., Federici S. (2023). Biotechnological Methods to Remove Microplastics: A Review. Environ. Chem. Lett..

[4] Ziani K., Ioniță-Mîndrican C.B., Mititelu M., Neacșu S.M., Negrei C., Moroșan E., Drăgănescu D., Preda O.T. (2023). Microplastics: A Real Global Threat for Environment and Food Safety: A State of the Art Review. Nutrients.

[5] Shetty S.S., D D., S H., Sonkusare S., Naik P.B., Kumari N S., Madhyastha H. (2023). Environmental Pollutants and Their Effects on Human Health. Heliyon.

[6] Matsumura Y., Kitabatake M., Kayano S.I., Ito T. (2023). Dietary Phenolic Compounds: Their Health Benefits and Association with the Gut Microbiota. Antioxidants.

[7] Bhaswant M., Shanmugam D.K., Miyazawa T., Abe C., Miyazawa T. (2023). Microgreens—A Comprehensive Review of Bioactive Molecules and Health Benefits. Molecules.

[8] de Araújo F.F., de Paulo Farias D., Neri-Numa I.A., Pastore G.M. (2021). Polyphenols and Their Applications: An Approach in Food Chemistry and Innovation Potential. Food Chem..

[9] Lucas-González R., Viuda-Martos M., Pérez-Alvarez J.A., Fernández-López J. (2018). In Vitro Digestion Models Suitable for Foods: Opportunities for New Fields of Application and Challenges. Food Res. Int..

[10] Sehrish A., Majeed I., Zongo E., Ayub H., Rasul H., Rahim M.A., AL-Asmari F. (2023). A Review on Various Extraction and Detection Methods of Bio-Functional Components from Microgreens: Food Applications and Health Properties. Int. J. Food Prop..

[11] Samuolienė G., Brazaitytė A., Viršilė A., Miliauskienė J., Vaštakaitė-Kairienė V., Duchovskis P. (2019). Nutrient Levels in Brassicaceae Microgreens Increase under Tailored Light-Emitting Diode Spectra. Front. Plant Sci..

[12] Arruda H.S., Neri-Numa I.A., Kido L.A., Maróstica Júnior M.R., Pastore G.M. (2020). Recent Advances and Possibilities for the Use of Plant Phenolic Compounds to Manage Ageing-Related Diseases. J. Funct. Foods.

[13] Pereira G.A., Arruda H.S., de Morais D.R., Peixoto Araujo N.M., Pastore G.M. (2020). Mutamba (*Guazuma ulmifolia* Lam.) Fruit as a Novel Source of Dietary Fibre and Phenolic Compounds. Food Chem..

[14] Singh A.K., Kim J.Y., Lee Y.S. (2022). Phenolic Compounds in Active Packaging and Edible Films/Coatings: Natural Bioactive Molecules and Novel Packaging Ingredients. Molecules.

[15] Câmara J.S., Albuquerque B.R., Aguiar J., Corrêa R.C.G., Gonçalves J.L., Granato D., Pereira J.A.M., Barros L., Ferreira I.C.F.R. (2021). Food Bioactive Compounds and Emerging Techniques for Their Extraction: Polyphenols as a Case Study. Foods.

[16] Rosales T.K.O., Fabi J.P. (2023). Valorization of Polyphenolic Compounds from Food Industry By-Products for Application in Polysaccharide-Based Nanoparticles. Front. Nutr..

[17] Wang X., Qi Y., Zheng H., Wang X., Qi Y., Zheng H. (2022). Dietary Polyphenol, Gut Microbiota, and Health Benefits. Antioxidants.

[18] Cheng H., Zhang D., Wu J., Liu J., Zhou Y., Tan Y., Feng W., Peng C. (2023). Interactions between Gut Microbiota and Polyphenols: A Mechanistic and Metabolomic Review. Phytomedicine.

[19] Bontzolis C.D., Dimitrellou D., Plioni I., Kandylis P., Soupioni M., Koutinas A.A., Kanellaki M. (2024). Effect of Solvents on Aniseed Aerial Plant Extraction Using Soxhlet and Ultrasound Methods, Regarding Antimicrobial Activity and Total Phenolic Content. Food Chem. Adv..

[20] Popova A., Mihaylova D. (2019). Antinutrients in Plant-Based Foods: A Review. Open Biotechnol. J..

[21] Nayik G.A., Gull A. (2020). Antioxidants in Fruits: Properties and Health Benefits.

[22] Rakariyatham K., Zhou D., Rakariyatham N., Shahidi F. (2020). Sapindaceae (*Dimocarpus longan* and *Nephelium lappaceum*) Seed and Peel by-Products: Potential Sources for Phenolic Compounds and Use as Functional Ingredients in Food and Health Applications. J. Funct. Foods.

[23] Singh N., Yadav S.S. (2022). A Review on Health Benefits of Phenolics Derived from Dietary Spices. Curr. Res. Food Sci..

[24] Wang Z., Li S., Ge S., Lin S. (2020). Review of Distribution, Extraction Methods, and Health Benefits of Bound Phenolics in Food Plants. J. Agric. Food Chem..

[25] Panja P. (2018). Green Extraction Methods of Food Polyphenols from Vegetable Materials. Curr. Opin. Food Sci..

[26] Zhang Y., Cai P., Cheng G., Zhang Y. (2022). A Brief Review of Phenolic Compounds Identified from Plants: Their Extraction, Analysis, and Biological Activity. Nat. Prod. Commun..

[27] Lin D., Xiao M., Zhao J., Li Z., Xing B., Li X., Kong M., Li L., Zhang Q., Liu Y. (2016). An Overview of Plant Phenolic Compounds and Their Importance in Human Nutrition and Management of Type 2 Diabetes. Molecules.

[28] Prabhu S., Molath A., Choksi H., Kumar S., Mehra R. (2021). Classifications of Polyphenols and Their Potential Application in Human Health and Diseases. Int. J. Physiol. Nutr. Phys. Educ..

[29] Durazzo A., Lucarini M., Souto E.B., Cicala C., Caiazzo E., Izzo A.A., Novellino E., Santini A. (2019). Polyphenols: A Concise Overview on the Chemistry, Occurrence, and Human Health. Phytother. Res..

[30] Kumar N., Goel N. (2019). Phenolic Acids: Natural Versatile Molecules with Promising Therapeutic Applications. Biotechnol. Rep..

[31] de Freitas Marinho L., Sganzerla W.G., Velasquez J.A., Gomes da Silva A.P., Rostagno M.A., Forster-Carneiro T. (2024). A Bibliometric Analysis of Phenolic Acids over the Last Five Years. Biocatal. Agric. Biotechnol..

[32] Piazzon A., Vrhovsek U., Masuero D., Mattivi F., Mandoj F., Nardini M. (2012). Antioxidant Activity of Phenolic Acids and Their Metabolites: Synthesis and Antioxidant Properties of the Sulfate Derivatives of Ferulic and Caffeic Acids and of the Acyl Glucuronide of Ferulic Acid. J. Agric. Food Chem..

[33] Cal M., Szakonyi Z., Pavlíková N. (2023). Caffeic Acid and Diseases—Mechanisms of Action. Int. J. Mol. Sci..

[34] Kiokias S., Proestos C., Oreopoulou V. (2020). Phenolic Acids of Plant Origin—A Review on Their Antioxidant Activity In Vitro (O/W Emulsion Systems) Along with Their in Vivo Health Biochemical Properties. Foods.

[35] Cosme P., Rodríguez A.B., Espino J., Garrido M. (2020). Plant Phenolics: Bioavailability as a Key Determinant of Their Potential Health-Promoting Applications. Antioxidants.

[36] Liga S., Paul C., Péter F. (2023). Flavonoids: Overview of Biosynthesis, Biological Activity, and Current Extraction Techniques. Plants.

[37] Tariq H., Asif S., Andleeb A., Hano C., Abbasi B.H. (2023). Flavonoid Production: Current Trends in Plant Metabolic Engineering and De Novo Microbial Production. Metabolites.

[38] Kuntorini E.M., Nugroho L.H., Maryani B., Nuringtyas T.R. (2019). Anatomical Structure, Flavonoid Content, and Antioxidant Activity of Rhodomyrtus Tomentosa Leaves and Fruits on Different Age and Maturity Level. Biodivers. J. Biol. Divers..

[39] Turatbekova A., Babamuradova L., Tasheva U., Saparbaeva N., Saibnazarova G., Turayeva M., Yakubov Y. (2023). A Brief Review on Biological and Chemical Activities of Flavonoids in Plants. E3S Web Conf..

[40] Abou Baker D.H. (2022). An Ethnopharmacological Review on the Therapeutical Properties of Flavonoids and Their Mechanisms of Actions: A Comprehensive Review Based on up to Date Knowledge. Toxicol. Rep..

[41] Khoo H.E., Azlan A., Tang S.T., Lim S.M. (2017). Anthocyanidins and Anthocyanins: Colored Pigments as Food, Pharmaceutical Ingredients, and the Potential Health Benefits. Food Nutr. Res..

[42] Bagniewska-Zadworna A., Barakat A., Łakomy P., Smoliński D.J., Zadworny M. (2014). Lignin and Lignans in Plant Defence: Insight from Expression Profiling of Cinnamyl Alcohol Dehydrogenase Genes during Development and Following Fungal Infection in *Populus*. Plant Sci..

[43] Cui Q., Du R., Liu M., Rong L. (2020). Lignans and Their Derivatives from Plants as Antivirals. Molecules.

[44] Calvo-Flores F.G., Dobado J.A., Isac-García J., Martin-Martinez F.J. (2015). Lignin and Lignans as Renewable Raw Materials: Chemistry, Technology and Applications.

[45] Consonni R., Ottolina G. (2022). NMR Characterization of Lignans. Molecules.

[46] Rodríguez-García C., Sánchez-Quesada C., Toledo E., Delgado-Rodríguez M., Gaforio J.J. (2019). Naturally Lignan-Rich Foods: A Dietary Tool for Health Promotion?. Molecules.

[47] Zhang J., Chen J., Liang Z., Zhao C. (2014). New Lignans and Their Biological Activities. Chem. Biodivers..

[48] Mendonça E.L.S.S., Xavier J.A., Fragoso M.B.T., Silva M.O., Escodro P.B., Oliveira A.C.M., Tucci P., Saso L., Goulart M.O.F. (2024). *E*-Stilbenes: General Chemical and Biological Aspects, Potential Pharmacological Activity Based on the Nrf2 Pathway. Pharmaceuticals.

[49] Teka T., Zhang L., Ge X., Li Y., Han L., Yan X. (2022). Stilbenes: Source Plants, Chemistry, Biosynthesis, Pharmacology, Application and Problems Related to Their Clinical Application-A Comprehensive Review. Phytochemistry.

[50] Al-Khayri J.M., Mascarenhas R., Harish H.M., Gowda Y., Lakshmaiah V.V., Nagella P., Al-Mssallem M.Q., Alessa F.M., Almaghasla M.I., Rezk A.A.S. (2023). Stilbenes, a Versatile Class of Natural Metabolites for Inflammation—An Overview. Molecules.

[51] Brown K., Theofanous D., Britton R.G., Aburido G., Pepper C., Sri Undru S., Howells L. (2024). Resveratrol for the Management of Human Health: How Far Have We Come? A Systematic Review of Resveratrol Clinical Trials to Highlight Gaps and Opportunities. Int. J. Mol. Sci..

[52] Paula A., Zomer L., Rodrigues C.A., Maldaner L. (2022). Piceatannol: A Natural Stilbene with a Broad Spectrum of Biological Activities. Res. Soc. Dev..

[53] Su X., Zhou D., Li N. (2022). Bioactive Stilbenes from Plants. Studies in Natural Products Chemistry.

[54] Tong Z., He W., Fan X., Guo A. (2022). Biological Function of Plant Tannin and Its Application in Animal Health. Front. Vet. Sci..

[55] Farha A.K., Yang Q.Q., Kim G., Li H.-B., Zhu F., Liu H.Y., Gan R.Y., Corke H. (2020). Tannins as an Alternative to Antibiotics. Food Biosci..

[56] Zeng X., Jiang W., Du Z., Kokini J.L. (2023). Encapsulation of Tannins and Tannin-Rich Plant Extracts by Complex Coacervation to Improve Their Physicochemical Properties and Biological Activities: A Review. Crit. Rev. Food Sci. Nutr..

[57] Fernández-Peña L., Matos M.J., López E. (2023). Recent Advances in Biologically Active Coumarins from Marine Sources: Synthesis and Evaluation. Mar. Drugs.

[58] Feng D., Zhang A., Yang Y., Yang P. (2020). Coumarin-Containing Hybrids and Their Antibacterial Activities. Arch. Pharm..

[59] Citarella A., Vittorio S., Dank C., Ielo L. (2024). Syntheses, Reactivity, and Biological Applications of Coumarins. Front. Chem..

[60] Ballin N.Z., Sørensen A.T. (2014). Coumarin Content in Cinnamon Containing Food Products on the Danish Market. Food Control.

[61] Krüger S., Winheim L., Morlock G.E. (2018). Planar Chromatographic Screening and Quantification of Coumarin in Food, Confirmed by Mass Spectrometry. Food Chem..

[62] Malik A., Khatkar A., Kakkar S. (2023). A Review on Pharmacological Activities of Vanillic Acid and Its Derivatives. Indo Glob. J. Pharm. Sci..

[63] Database on Polyphenol Content in Foods—Phenol-Explorer. http://phenol-explorer.eu/.

[64] Janabi A.H.W., Kamboh A.A., Saeed M., Lu X., BiBi J., Majeed F., Naveed M., Mughal M.J., Korejo N.A., Kamboh R. (2020). Flavonoid-Rich Foods (FRF): A Promising Nutraceutical Approach against Lifespan-Shortening Diseases. Iran. J. Basic Med. Sci..

[65] Reinisalo M., Kårlund A., Koskela A., Kaarniranta K., Karjalainen R.O. (2015). Polyphenol Stilbenes: Molecular Mechanisms of Defence against Oxidative Stress and Aging-Related Diseases. Oxid. Med. Cell. Longev..

[66] Benbouguerra N., Hornedo-Ortega R., Garcia F., El Khawand T., Saucier C., Richard T. (2021). Stilbenes in Grape Berries and Wine and Their Potential Role as Anti-Obesity Agents: A Review. Trends Food Sci. Technol..

[67] Ojo M.A. (2022). Tannins in Foods: Nutritional Implications and Processing Effects of Hydrothermal Techniques on Underutilized Hard-to-Cook Legume Seeds—A Review. Prev. Nutr. Food Sci..

[68] Fraga-Corral M., Otero P., Echave J., Garcia-Oliveira P., Carpena M., Jarboui A., Nuñez-Estevez B., Simal-Gandara J., Prieto M.A. (2021). By-Products of Agri-Food Industry as Tannin-Rich Sources: A Review of Tannins’ Biological Activities and Their Potential for Valorization. Foods.

[69] Lončar M., Jakovljević M., Šubarić D., Pavlić M., Služek V.B., Cindrić I., Molnar M. (2020). Coumarins in Food and Methods of Their Determination. Foods.

[70] Arigò A., Rigano F., Russo M., Trovato E., Dugo P., Mondello L. (2021). Dietary Intake of Coumarins and Furocoumarins through Citrus Beverages: A Detailed Estimation by a HPLC-MS/MS Method Combined with the Linear Retention Index System. Foods.

[71] Bai Y., Li D., Zhou T., Qin N., Li Z., Yu Z., Hua H. (2016). Coumarins from the Roots of *Angelica dahurica* with Antioxidant and Antiproliferative Activities. J. Funct. Foods.

[72] Peterson J., Dwyer J., Adlercreutz H., Scalbert A., Jacques P., McCullough M.L. (2010). Dietary Lignans: Physiology and Potential for Cardiovascular Disease Risk Reduction. Nutr. Rev..

[73] Yan B., Chen Z.S., Hu Y., Yong Q. (2021). Insight in the Recent Application of Polyphenols from Biomass. Front. Bioeng. Biotechnol..

[74] Deshmukh R.K., Gaikwad K.K. (2022). Natural Antimicrobial and Antioxidant Compounds for Active Food Packaging Applications. Biomass Convers. Biorefin..

[75] Siddiqui S.A., Khan S., Mehdizadeh M., Bahmid N.A., Adli D.N., Walker T.R., Perestrelo R., Câmara J.S. (2023). Phytochemicals and Bioactive Constituents in Food Packaging—A Systematic Review. Heliyon.

[76] de Paulo Farias D., Neri-Numa I.A., de Araújo F.F., Pastore G.M. (2020). A Critical Review of Some Fruit Trees from the Myrtaceae Family as Promising Sources for Food Applications with Functional Claims. Food Chem..

[77] Chawla R., Sivakumar S., Kaur H. (2021). Antimicrobial Edible Films in Food Packaging: Current Scenario and Recent Nanotechnological Advancements- a Review. Carbohydr. Polym. Technol. Appl..

[78] Rahman S.M.A., Nassef A.M., Al-Dhaifallah M., Abdelkareem M.A., Rezk H. (2020). The Effect of a New Coating on the Drying Performance of Fruit and Vegetables Products: Experimental Investigation and Artificial Neural Network Modeling. Foods.

[79] Salehi F., Ghazvineh S., Inanloodoghouz M. (2023). Effects of Edible Coatings and Ultrasonic Pretreatment on the Phenolic Content, Antioxidant Potential, Drying Rate, and Rehydration Ratio of Sweet Cherry. Ultrason. Sonochem..

[80] Martillanes S., Rocha-Pimienta J., Cabrera-Bañegil M., Martín-Vertedor D., Delgado-Adámez J. (2017). Application of Phenolic Compounds for Food Preservation: Food Additive and Active Packaging. Phenolic Compounds—Biological Activity.

[81] Padavic-Callaghan K. (2023). Colour-Changing Wrap Tells You When Food Has Spoiled. New Sci..

[82] Leong H.Y., Show P.L., Lim M.H., Ooi C.W., Ling T.C. (2018). Natural Red Pigments from Plants and Their Health Benefits: A Review. Food Rev. Int..

[83] Al-Tayyar N.A., Youssef A.M., Al-hindi R. (2020). Antimicrobial Food Packaging Based on Sustainable Bio-Based Materials for Reducing Foodborne Pathogens: A Review. Food Chem..

[84] Shahid-ul-Islam, Mohammad F. (2016). Potent Polyphenolic Natural Colorants Derived from Plants as Eco-Friendly Raw Materials for the Dyeing Industry. Green Fashion.

[85] Albuquerque B.R., Oliveira M.B.P.P., Barros L., Ferreira I.C.F.R. (2021). Could Fruits Be a Reliable Source of Food Colorants? Pros and Cons of These Natural Additives. Crit. Rev. Food Sci. Nutr..

[86] Li N., Wang Q., Zhou J., Li S., Liu J., Chen H. (2022). Insight into the Progress on Natural Dyes: Sources, Structural Features, Health Effects, Challenges, and Potential. Molecules.

[87] Bahreini Z., Abedi M., Fateh D.S., Nazemi A.H. (2021). Food Colorants, Requirements and Approaches. J. Stud. Color World.

[88] Weiss V., Okun Z., Shpigelman A. (2023). Tackling the Safety and Health Effects of Food Colorants. Food Saf. Health.

[89] Saefudin E.B. (2023). Utilization of Tannin as Renewable Natural Pigment in the Culture of Indonesian Batik Fabrics: A Review. J. Posit. Sch. Psychol..

[90] Das A.K., Islam M.N., Faruk M.O., Ashaduzzaman M., Dungani R. (2020). Review on Tannins: Extraction Processes, Applications and Possibilities. S. Afr. J. Bot..

[91] Giusti M.M., Miyagusuku-Cruzado G., Wallace T.C. (2023). Flavonoids as Natural Pigments. Handbook of Natural Colorants.

[92] Vega E.N., Ciudad-Mulero M., Fernández-Ruiz V., Barros L., Morales P. (2023). Natural Sources of Food Colorants as Potential Substitutes for Artificial Additives. Foods.

[93] Lang Y., Gao N., Zang Z., Meng X., Lin Y., Yang S., Yang Y., Jin Z., Li B. (2024). Classification and Antioxidant Assays of Polyphenols: A Review. J. Future Foods.

[94] Bešlo D., Golubić N., Rastija V., Agić D., Karnaš M., Šubarić D., Lučić B. (2023). Antioxidant Activity, Metabolism, and Bioavailability of Polyphenols in the Diet of Animals. Antioxidants.

[95] Chojnacka K., Skrzypczak D., Izydorczyk G., Witek-Krowiak A., Mikula K., Szopa D. (2021). Antiviral Properties of Polyphenols from Plants. Foods.

[96] Richart S.M., Li Y.L., Mizushina Y., Chang Y.Y., Chung T.Y., Chen G.H., Tzen J.T.C., Shia K.S., Hsu W.L. (2018). Synergic Effect of Curcumin and Its Structural Analogue (Monoacetylcurcumin) on Anti-Influenza Virus Infection. J. Food Drug Anal..

[97] Nagai E., Iwai M., Koketsu R., Sogabe R., Morimoto R., Suzuki Y., Ohta Y., Okuno Y., Ohshima A., Enomoto T. (2018). Inhibition of Influenza Virus Replication by Adlay Tea. J. Sci. Food Agric..

[98] Zhang M., Wu Q., Chen Y., Duan M., Tian G., Deng X., Sun Y., Zhou T., Zhang G., Chen W. (2018). Inhibition of Proanthocyanidin A2 on Porcine Reproductive and Respiratory Syndrome Virus Replication in Vitro. PLoS ONE.

[99] Montenegro-Landívar M.F., Tapia-Quirós P., Vecino X., Reig M., Valderrama C., Granados M., Cortina J.L., Saurina J. (2021). Polyphenols and Their Potential Role to Fight Viral Diseases: An Overview. Sci. Total Environ..

[100] Manso T., Lores M., de Miguel T. (2022). Antimicrobial Activity of Polyphenols and Natural Polyphenolic Extracts on Clinical Isolates. Antibiotics.

[101] Efenberger-Szmechtyk M., Nowak A., Czyzowska A. (2021). Plant Extracts Rich in Polyphenols: Antibacterial Agents and Natural Preservatives for Meat and Meat Products. Crit. Rev. Food Sci. Nutr..

[102] Chen X., Lan W., Xie J. (2024). Natural Phenolic Compounds: Antimicrobial Properties, Antimicrobial Mechanisms, and Potential Utilization in the Preservation of Aquatic Products. Food Chem..

[103] Ecevit K., Barros A.A., Silva J.M., Reis R.L. (2022). Preventing Microbial Infections with Natural Phenolic Compounds. Future Pharmacol..

[104] Takó M., Kerekes E.B., Zambrano C., Kotogán A., Papp T., Krisch J., Vágvölgyi C. (2020). Plant Phenolics and Phenolic-Enriched Extracts as Antimicrobial Agents against Food-Contaminating Microorganisms. Antioxidants.

[105] Longevity O.M.A.C. (2024). Retracted: Fermented Carrot Pulp Regulates the Dysfunction of Murine Intestinal Microbiota. Oxidative Med. Cell. Longev..

[106] Yang F., Chen C., Ni D., Yang Y., Tian J., Li Y., Chen S., Ye X., Wang L. (2023). Effects of Fermentation on Bioactivity and the Composition of Polyphenols Contained in Polyphenol-Rich Foods: A Review. Foods.

[107] Mihaylova D., Popova A., Goranova Z., Doykina P. (2022). Development of Healthy Vegan Bonbons Enriched with Lyophilized Peach Powder. Foods.

[108] Petkova T., Doykina P., Alexieva I., Mihaylova D., Popova A. (2022). Characterization of Fruit Sorbet Matrices with Added Value from *Zizyphus jujuba* and *Stevia rebaudiana*. Foods.

[109] Rutkowska J., Antoniewska A., Martinez-Pineda M., Nawirska-Olszańska A., Zbikowska A., Baranowski D. (2020). Black Chokeberry Fruit Polyphenols: A Valuable Addition to Reduce Lipid Oxidation of Muffins Containing Xylitol. Antioxidants.

[110] Kindernay L., Ferenczyová K., Farkašová V., Dulová U., Strapec J., Barteková M. (2023). Beneficial Effects of Polyphenol-Rich Food Oils in Cardiovascular Health and Disease. Rev. Cardiovasc. Med..

[111] Livingstone K.M., Ramos-Lopez O., Pérusse L., Kato H., Ordovas J.M., Martínez J.A. (2022). Precision Nutrition: A Review of Current Approaches and Future Endeavors. Trends Food Sci. Technol..

[112] Firth J., Gangwisch J.E., Borisini A., Wootton R.E., Mayer E.A. (2020). Food for Thought 2020: Food and Mood: How Do Diet and Nutrition Affect Mental Wellbeing?. BMJ.

[113] Vallgårda S. (2011). Why the Concept “Lifestyle Diseases” Should Be Avoided. Scand. J. Public Health.

[114] Lassale C., Batty G.D., Baghdadli A., Jacka F., Sánchez-Villegas A., Kivimäki M., Akbaraly T. (2019). Healthy Dietary Indices and Risk of Depressive Outcomes: A Systematic Review and Meta-Analysis of Observational Studies. Mol. Psychiatry.

[115] Micek A., Owczarek M., Jurek J., Guerrera I., Torrisi S.A., Grosso G., Alshatwi A.A., Godos J. (2022). Anthocyanin-Rich Fruits and Mental Health Outcomes in an Italian Cohort. J. Berry Res..

[116] Lin K., Li Y., Toit E.D., Wendt L., Sun J. (2021). Effects of Polyphenol Supplementations on Improving Depression, Anxiety, and Quality of Life in Patients with Depression. Front. Psychiatry.

[117] D’Angelo S. (2023). Diet and Aging: The Role of Polyphenol-Rich Diets in Slow Down the Shortening of Telomeres: A Review. Antioxidants.

[118] Remigante A., Spinelli S., Straface E., Gambardella L., Russo M., Cafeo G., Caruso D., Falliti G., Dugo P., Dossena S. (2023). Mechanisms Underlying the Anti-Aging Activity of Bergamot (Citrus Bergamia) Extract in Human Red Blood Cells. Front. Physiol..

[119] Meccariello R., D’Angelo S. (2021). Impact of Polyphenolic-Food on Longevity: An Elixir of Life. An Overview. Antioxidants.

[120] Buettner D., Skemp S. (2016). Blue Zones: Lessons From the World’s Longest Lived. Am. J. Lifestyle Med..

[121] Restani P., Colombo F., Biella S., Bani C., Mercogliano F., Di Lorenzo C. (2022). Diet, Polyphenols, and Human Evolution. Appl. Sci..

[122] Finicelli M., Di Salle A., Galderisi U., Peluso G. (2022). The Mediterranean Diet: An Update of the Clinical Trials. Nutrients.

[123] Guasch-Ferré M., Willett W.C. (2021). The Mediterranean Diet and Health: A Comprehensive Overview. J. Intern. Med..

[124] Delgado A.M., Vaz Almeida M.D., Parisi S. (2016). Chemistry of the Mediterranean Diet.

[125] Schlesinger S., Neuenschwander M., Schwedhelm C., Hoffmann G., Bechthold A., Boeing H., Schwingshackl L. (2019). Food Groups and Risk of Overweight, Obesity, and Weight Gain: A Systematic Review and Dose-Response Meta-Analysis of Prospective Studies. Adv. Nutr..

[126] Kanner J. (2023). Food Polyphenols as Preventive Medicine. Antioxidants.

[127] Zekrumah M., Begua P., Razak A., Wahab J., Moffo N., Ivane A., Oman M., Elrashied H., Zou X., Zhang D. (2023). Role of Dietary Polyphenols in Non-Communicable Chronic Disease Prevention, and Interactions in Food Systems: An Overview. Nutrition.

[128] Kanner J., Selhub J., Shpaizer A., Rabkin B., Shacham I., Tirosh O. (2017). Redox Homeostasis in Stomach Medium by Foods: The Postprandial Oxidative Stress Index (POSI) for Balancing Nutrition and Human Health. Redox Biol..

[129] Kanner J. (2007). Dietary Advanced Lipid Oxidation Endproducts Are Risk Factors to Human Health. Mol. Nutr. Food Res..

[130] Xie F., Yang W., Xing M., Zhang H., Ai L. (2023). Natural Polyphenols-Gut Microbiota Interactions and Effects on Glycolipid Metabolism via Polyphenols-Gut-Brain Axis: A State-of-the-Art Review. Trends Food Sci. Technol..

[131] Bačić A., Gavrilović J., Rajilić S.M. (2023). Polyphenols as a New Class of Prebiotics for Gut Microbiota Manipulation. Arh. Farm..

[132] Plamada D., Vodnar D.C. (2022). Polyphenols—Gut Microbiota Interrelationship: A Transition to a New Generation of Prebiotics. Nutrients.

[133] Domínguez-Avila J.A., Villa-Rodriguez J.A., Montiel-Herrera M., Pacheco-Ordaz R., Roopchand D.E., Venema K., González-Aguilar G.A. (2021). Phenolic Compounds Promote Diversity of Gut Microbiota and Maintain Colonic Health. Dig. Dis. Sci..

[134] Mithul Aravind S., Wichienchot S., Tsao R., Ramakrishnan S., Chakkaravarthi S. (2021). Role of Dietary Polyphenols on Gut Microbiota, Their Metabolites and Health Benefits. Food Res. Int..

[135] Han D., Wu Y., Lu D., Pang J., Hu J., Zhang X., Wang Z., Zhang G., Wang J. (2023). Polyphenol-Rich Diet Mediates Interplay between Macrophage-Neutrophil and Gut Microbiota to Alleviate Intestinal Inflammation. Cell Death Dis..

[136] Martínez-Montoro J.I., Quesada-Molina M., Gutiérrez-Repiso C., Ruiz-Limón P., Subiri-Verdugo A., Tinahones F.J., Moreno-Indias I. (2022). Effect of Moderate Consumption of Different Phenolic-Content Beers on the Human Gut Microbiota Composition: A Randomized Crossover Trial. Antioxidants.

[137] Rodríguez-Daza M.C., de Vos W.M. (2023). Polyphenols as Drivers of a Homeostatic Gut Microecology and Immuno-Metabolic Traits of Akkermansia Muciniphila: From Mouse to Man. Int. J. Mol. Sci..

[138] Vita A.A., Roberts K.M., Gundersen A., Farris Y., Zwickey H., Bradley R., Weir T.L. (2024). Relationships between Habitual Polyphenol Consumption and Gut Microbiota in the INCLD Health Cohort. Nutrients.

[139] Duda-Chodak A., Tarko T. (2023). Possible Side Effects of Polyphenols and Their Interactions with Medicines. Molecules.

[140] Shaito A., Posadino A.M., Younes N., Hasan H., Halabi S., Alhababi D., Al-Mohannadi A., Abdel-Rahman W.M., Eid A.H., Nasrallah G.K. (2020). Potential Adverse Effects of Resveratrol: A Literature Review. Int. J. Mol. Sci..

[141] Kadac-Czapska K., Knez E., Gierszewska M., Olewnik-Kruszkowska E., Grembecka M. (2023). Microplastics Derived from Food Packaging Waste—Their Origin and Health Risks. Materials.

[142] Di Fiore C., Carriera F., Russo M.V., Avino P. (2023). Are Microplastics a Macro Issue? A Review on the Sources of Contamination, Analytical Challenges and Impact on Human Health of Microplastics in Food. Foods.

[143] Wang Y., Wang M., Wang Q., Wang T., Zhou Z., Mehling M., Guo T., Zou H., Xiao X., He Y. (2023). Flowthrough Capture of Microplastics through Polyphenol-Mediated Interfacial Interactions on Wood Sawdust. Adv. Mater..

[144] Kwon H.J., Hidayaturrahman H., Peera S.G., Lee T.G. (2022). Elimination of Microplastics at Different Stages in Wastewater Treatment Plants. Water.

[145] Rani M., Ducoli S., Depero L.E., Prica M., Tubić A., Ademovic Z., Morrison L., Federici S. (2023). A Complete Guide to Extraction Methods of Microplastics from Complex Environmental Matrices. Molecules.

[146] Stan D., Enciu A.M., Mateescu A.L., Ion A.C., Brezeanu A.C., Stan D., Tanase C. (2021). Natural Compounds with Antimicrobial and Antiviral Effect and Nanocarriers Used for Their Transportation. Front. Pharmacol..

[147] Urban-Chmiel R., Marek A., Stępień-Pyśniak D., Wieczorek K., Dec M., Nowaczek A., Osek J. (2022). Antibiotic Resistance in Bacteria-A Review. Antibiotics.

[148] Rodrigues D.B., Veríssimo L., Finimundy T., Rodrigues J., Oliveira I., Gonçalves J., Fernandes I.P., Barros L., Heleno S.A., Calhelha R.C. (2023). Chemical and Bioactive Screening of Green Polyphenol-Rich Extracts from Chestnut By-Products: An Approach to Guide the Sustainable Production of High-Added Value Ingredients. Foods.

[149] Palos-Hernández A., Gutiérrez Fernández M.Y., Escuadra Burrieza J., Pérez-Iglesias J.L., González-Paramás A.M. (2022). Obtaining Green Extracts Rich in Phenolic Compounds from Underexploited Food By-Products Using Natural Deep Eutectic Solvents. Opportunities and Challenges. Sustain. Chem. Pharm..

[150] Marisa Ribeiro A., Estevinho B.N., Rocha F. (2020). Microencapsulation of Polyphenols—The Specific Case of the Microencapsulation of *Sambucus Nigra* L. Extracts—A Review. Trends Food Sci. Technol..

